# Outpatient red blood cell transfusion payments among patients on chronic dialysis

**DOI:** 10.1186/1471-2369-13-145

**Published:** 2012-11-02

**Authors:** Matthew Gitlin, J Andrew Lee, David M Spiegel, Jeffrey L Carson, Xue Song, Brian S Custer, Zhun Cao, Katherine A Cappell, Helen V Varker, Shaowei Wan, Akhtar Ashfaq

**Affiliations:** 1 Amgen, Inc., One Amgen Center Drive, Thousand Oaks, CA, USA; 2 University of Colorado, Denver, CO, USA; 3 UMDNJ-Robert Wood Johnson Medical School, New Brunswick, NJ, USA; 4 Truven Health Analytics, Cambridge, MA, USA; 5 Blood Systems Research Institute, San Francisco, CA, USA

**Keywords:** Dialysis, Red blood cell transfusions, Payers, Cost

## Abstract

**Background:**

Payments for red blood cell (RBC) transfusions are separate from US Medicare bundled payments for dialysis-related services and medications. Our objective was to examine the economic burden for payers when chronic dialysis patients receive outpatient RBC transfusions.

**Methods:**

Using Truven Health MarketScan® data (1/1/02-10/31/10) in this retrospective micro-costing economic analysis, we analyzed data from chronic dialysis patients who underwent at least 1 outpatient RBC transfusion who had at least 6 months of continuous enrollment prior to initial dialysis claim and at least 30 days post-transfusion follow-up. A conceptual model of transfusion-associated resource use based on current literature was employed to estimate outpatient RBC transfusion payments. Total payments per RBC transfusion episode included screening/monitoring (within 3 days), blood acquisition/administration (within 2 days), and associated complications (within 3 days for acute events; up to 45 days for chronic events).

**Results:**

A total of 3283 patient transfusion episodes were included; 56.4% were men and 40.9% had Medicare supplemental insurance. Mean (standard deviation [SD]) age was 60.9 (15.0) years, and mean Charlson comorbidity index was 4.3 (2.5). During a mean (SD) follow-up of 495 (474) days, patients had a mean of 2.2 (3.8) outpatient RBC transfusion episodes. Mean/median (SD) total payment per RBC transfusion episode was $854/$427 ($2,060) with 72.1% attributable to blood acquisition and administration payments. Complication payments ranged from mean (SD) $213 ($168) for delayed hemolytic transfusion reaction to $19,466 ($15,424) for congestive heart failure.

**Conclusions:**

Payments for outpatient RBC transfusion episodes were driven by blood acquisition and administration payments. While infrequent, transfusion complications increased payments substantially when they occurred.

## Background

Anemia is common in end-stage renal disease (ESRD) and results from reduction of erythropoietin production [1]. Prior to the development of pharmacologic treatments for anemia, red blood cell (RBC) transfusions were the mainstay of anemia treatment, and approximately 55% to 60% of dialysis patients received RBC transfusions to avoid severe anemia [2,3]. RBC transfusions are associated with a variety of complications, including hemolytic and non-hemolytic transfusion reactions, infections, transfusion-related acute lung injury (TRALI), transfusion-associated circulatory overload (TACO), and hyperkalemia [3-10]. In 2009, the rate of RBC transfusion-associated adverse reactions across all disease states was reported as approximately 0.25% [11]. However, the rates of RBC transfusion-related complications may be higher among chronic dialysis patients because of their significant comorbid disease severity and concerns about patients’ fluid overload. Furthermore, while the overall rates of these complications may be low, their outcomes can be severe (hospitalization or death) and their associated costs are high [12-14].

The use of RBC transfusion as a treatment for anemia declined dramatically after the approval of the first erythropoiesis-stimulating agent (ESA) in 1989 [15,16]. In the 1995 annual report of the United States Renal Data System (USRDS), the reported rate of outpatient RBC transfusion in hemodialysis patients dropped from 16% in 1989 to 2% by 1993 [17]. The overall rate of RBC transfusions (per 1000 patient-years) in the hemodialysis setting decreased by about half from 535.33 in 1992 to 263.65 in 2005 [16]. About 83% to 94% of chronic dialysis patients now use an ESA to treat chronic anemia [18]. With the newly implemented Medicare Prospective Payment System (PPS) for ESRD patients, reimbursement to providers is capitated to include dialysis and separately billable medications and services (ie, ESAs, iron, dialysis supplies, lab tests) but does not include blood and blood products. Since RBC transfusion use in ESRD patients on dialysis could potentially increase, it is important to understand transfusion-associated payments and outcomes in this patient population.

Incomplete accounting of payments related to RBC transfusion administration may provide misleading information to policy makers determining reimbursement policy in a healthcare system such as Medicare. The purpose of this retrospective claims analysis study was to use a micro-costing approach to examine the economic burden for payers when chronic dialysis patients receive outpatient RBC transfusions. This study will assist in quantifying the economic impact to payers of RBC transfusions as they are tracked within many of the PPS surveillance programs and will inform future studies examining RBC transfusion and associated payments in Medicare claims data.

## Methods

### Data sources

The Truven Health MarketScan® Commercial Claims and Encounter and Medicare Supplemental and Coordination of Benefits Databases were used for this study. These databases are constructed from privately insured paid medical and prescription drug claims. The MarketScan Commercial Database contains the inpatient, outpatient, and outpatient prescription drug experience of approximately 30 million employees and their dependents (in 2010) covered under a variety of fee-for-service plans, managed care health plans, and indemnity plans. In addition, the MarketScan Medicare Database contains the healthcare experience of approximately 3.42 million retirees (in 2010) with Medicare supplemental insurance paid for by employers. Medicare-covered portion of payment, employer-paid portion, and patient-paid portion are included in this database. The MarketScan Commercial and Medicare Databases provide detailed cost, use, and outcomes data for healthcare services performed in both inpatient and outpatient settings. The medical claims are linked to outpatient prescription-drug claims and person-level enrollment data through the use of unique enrollee identifiers. All personal identifiers were removed.

### Transfusion episode inclusion criteria

All analyses in this study were performed at the level of the transfusion episode. Eligible patients were first identified using the following criteria: inclusion in the MarketScan Commercial or Medicare Databases with ≥ 2 claims (to ensure that they were treated for chronic disease) for chronic dialysis ≥ 30 days apart and within 365 days between January 1, 2002, and October 31, 2010 (codes used to identify chronic dialysis claims are listed in Additional file 1: Table S1); had ≥ 6 months of continuous enrollment prior to first chronic dialysis claim to measure baseline clinical characteristics; had ≥ 1 outpatient RBC blood transfusion on or after date of first chronic dialysis claim through January 31, 2011 (codes used to identify RBC blood transfusion claims are listed in Additional file 1: Table S2); had no terminating events (defined as end of continuous enrollment, end of MarketScan data [January 31, 2011], death, or kidney transplant) between first chronic dialysis claim and first outpatient RBC transfusion; and had ≥ 30 days of continuous enrollment after the first RBC transfusion to identify and measure subsequent RBC transfusion-related complications. RBC transfusions were identified using revenue codes (UB-04), Current Procedural Terminology (CPT), and Healthcare Common Procedure Coding System (HCPCS) codes (codes used to identify transfusion-related complications claims are listed in Additional file 1: Table S3).

**Figure 1:**
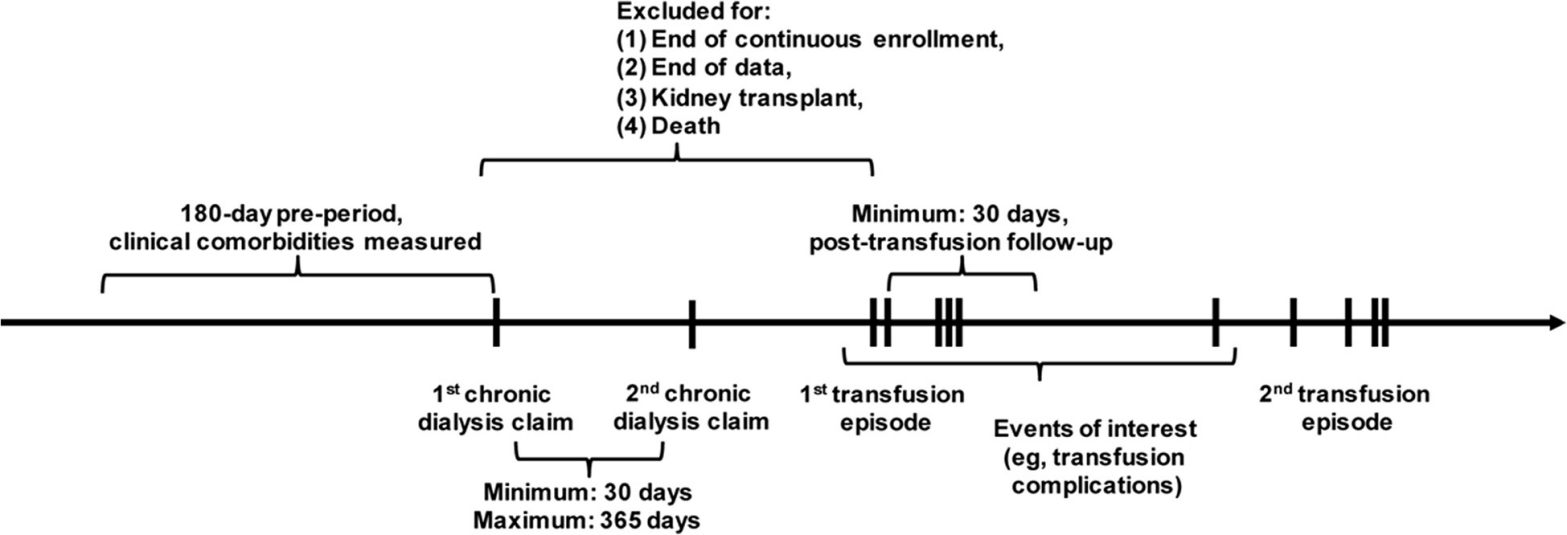
Patient selection time frame.

RBC transfusion–related complications that could be identified in the coded dataset and could be reasonably attributed to a transfusion episode (based on medical literature and expert clinical opinion) included febrile non-hemolytic transfusion reaction, air embolism, or phlebitis; acute hemolytic transfusion reaction; allergic reaction; TRALI; TACO; delayed hemolytic transfusion reaction; congestive heart failure (CHF); and hyperkalemia. To increase the likelihood that CHF or hyperkalemia complications were directly related to the RBC transfusion episode (rather than a pre-existing condition that coincided with the transfusion), patients with a history of CHF or hyperkalemia during the pre-index period were excluded from the complication cost analyses (but not from overall cost analysis). Because of the short follow-up period post–transfusion episode, we could not collect information on longer-term complications that might require more time to develop or be diagnosed (e.g., transfusion-related infections and iron overload). A summary of the patient selection and transfusion episode time frame is presented in Figure 1.

### Variable definition and time frame

Demographic information was collected on the date of the first chronic dialysis claim. Clinical characteristics were measured during the 180 days prior to the first chronic dialysis claim. Because some patients had multiple RBC transfusion claims within a short time frame, we combined individual claims within 3 days of each other into a transfusion episode, which was the unit of observation for this study. Because pre- and post-transfusion screening and monitoring payments could not be differentiated for patients with more than 1 transfusion claim within an episode, we combined screening and monitoring payments 3 days prior to and 3 days post–transfusion episode. Blood acquisition and administration payments were examined from transfusion episode date to 2 days post–RBC transfusion episode.

**Figure 2:**
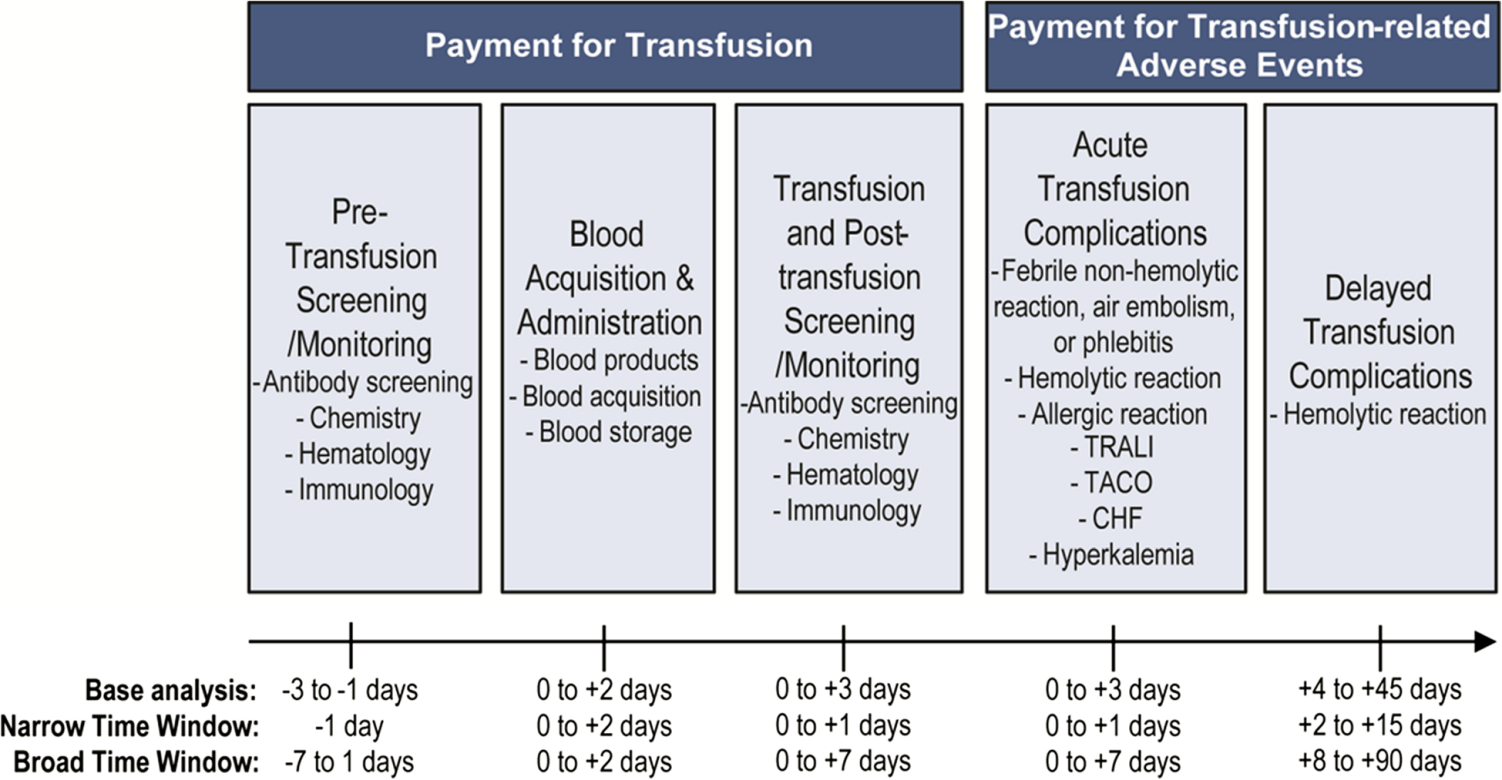
**Transfusion episode time frame.** Abbreviations: CHF, congestive heart failure; TACO, transfusion-associated circulatory overload; TRALI, transfusion-related acute lung injury.

With the exception of delayed hemolytic transfusion reactions, all complications were identified up to 0 to 3 days post–RBC transfusion episode. Hemolytic transfusion reactions were identified 4 to 45 days post–RBC transfusion episode (Figure 2). If a claim for RBC transfusion–related complication was linked to ≥ 1 RBC transfusion episode, it was linked to the earliest episode. If a claim for a complication could not be linked to an RBC transfusion episode based on the described time windows, the claim was not included in the analyses.

### Micro-costing/component analysis approach

We used a micro-costing approach to measure cost based on the components of resource units and their payment values [19-21]. We included payments for blood acquisition, transfusion administration, RBC transfusion–related lab tests, and transfusion complications [21]. For the base-case analysis, we calculated component payments for (1) RBC transfusion screening and monitoring, (2) blood acquisition and administration, and (3) transfusion-related complication, which were summed to calculate (4) total payment for each RBC transfusion episode.

We conducted subgroup analyses for (1) patients with an acute bleed or surgery during the 180 days prior to initial dialysis claim, (2) patients with cancer or blood disease during the 180 days prior to initial dialysis claim, and (3) patients who experienced an RBC transfusion-related complication, by type of complication. We also performed sensitivity analyses by excluding cost outliers (RBC transfusion episodes with the top 1% of blood acquisition and administration costs or those with costs equal to $0 were excluded), varying the payment time frames (using both a narrow and broad time window [defined in Figure 2] in identifying and defining payment claims to RBC transfusion episodes), and estimating mean payment per unit of blood based on a blood acquisition and administration claim analysis.

## Results

### Patient sample

From an initial sample of 105,260 patients with ≥ 2 chronic dialysis claims, we had a final sample of 3,283 chronic dialysis patients who met all of the selection criteria. Requiring 6 months of pre-index data and ≥ 1 outpatient RBC transfusion contributed to the greatest loss of subjects. Among the 3,283 chronic dialysis patients, there were 7,049 outpatient RBC transfusion episodes used in the micro-costing analyses. Mean (standard deviation [SD]) patient follow-up was 494.76 (474.19) days.

### Patient demographics and clinical characteristics

Patients’ demographic and clinical characteristics are presented in Table 1. Mean (SD) age was 60.9 (15.0) years, 56.4% of patients were men, and 40.9% of patients had Medicare supplemental insurance. The three most frequent comorbidities were hypertension (93.9%), diabetes (50.6%), and CHF (35.6%). Hemodialysis was performed in 60.8% of patients, peritoneal dialysis in 6.3%, and type of dialysis was unknown in 33.4%. Patients experienced a mean 2.15 transfusion episodes during the follow-up period. Transfusion was administered at outpatient hospital facilities in 82.0%, ESRD facilities in 9.4%, hospital emergency rooms in 2.6%, and unknown in 6%.

**Table 1:** Demographic and clinical characteristics

	**Patients**
	**N = 3,283**^**a**^
Age, mean years (SD)	60.9 (15.0)
Sex, n male (%)	1,850 (56.4)
Geographic region, n (%)	
Northeast	253 (7.7)
North Central	975 (29.7)
South	1,459 (44.4)
West	585 (17.8)
Unknown	11 (0.3)
Payer, n (%)	
Commercial	1,941 (59.1)
Medicare	1,342 (40.9)
Deyo Charlson comorbidity index, mean score (SD)	4.32 (2.45)
Comorbid conditions, n (%)	
Hypertension	3,084 (93.9)
Diabetes	1,662 (50.6)
CHF	1,167 (35.6)
Acute bleeding	748 (22.8)
Surgery	719 (21.9)
Cancer	680 (20.7)
COPD	460 (14.0)
Hyperkalemia	423 (12.9)
Dialysis modality, n (%)	
Hemodialysis	1,997 (60.8)
Peritoneal dialysis	207 (6.3)
Unknown	1,095 (33.4)
Transfusion episodes with ≥ 30 days follow-up, mean number (SD)	2.15 (3.78)
Length of follow-up, mean days (SD)	494.76 (474.19)

^a^Chronic dialysis patients with ≥ 1 outpatient red blood cell transfusion episode.

*CHF*, congestive heart failure; *COPD*, chronic obstructive pulmonary disease; *SD*, standard deviation.

### Red blood cell transfusion episode payments

The component and total payments for RBC blood transfusion episodes are presented in Table 2. Mean (SD) total payment per RBC transfusion episode was $854 ($2,060). The median payment was $427 (25th percentile, $53; 75th percentile, $1071), suggesting that payments were not normally distributed. The largest component (72.0%) of the total payment was blood acquisition and administration (mean, $615; SD, $1,237; median, $289). Pre- and post-transfusion screening and monitoring component payments represented 22.6% of the total payment (mean, $193; SD, $616; median, $34). Payments for complications when averaged across all RBC transfusion episodes were relatively low (mean, $75; SD, $1,317; median, $0) but individual episode payments ranged from mean (SD) $213 ($168) for delayed hemolytic transfusion reaction to $19,466 ($15,424) for CHF. When evaluating total payments by primary payer type, the mean payments were similar. Mean payments (median; SD) per RBC blood transfusion episode were $855 ($388; $2,728) and $853 ($457; $1,428) for Medicare primary and commercial primary, respectively.

**Table 2:** Base-case payment per red blood cell transfusion episode

**Payments and Events**	**All patients**^**a**^**(N= 3,283); All episodes (N = 7,049)**					**Min**	**Max**
	**Mean**	**SD**	**Median**	**25th percentile**	**75th percentile**		
**Average payment per episode**							
**All payers**							
Transfusion screening/monitoring	$193	$616	$34	$0	$189	$0	$22,673
Blood acquisition and administration	$615	$1,237	$289	$11	$801	$0	$30,962
Transfusion complications	$75	$1,317	$0	$0	$0	$0	$61,059
**TOTAL screening, transfusion and complication payments**	**$854**	**$2,060**	**$427**	**$53**	**$1,065**	**$0**	**$74,452**
**Number of services per episode**							
Transfusion screening/monitoring	2.87	2.93	2.00	0.09	5.00	0.00	20.00
Blood acquisition and administration	2.04	1.26	2.00	1.00	3.00	1.00	12.83

^a^Patients with ≥ 1 outpatient red blood cell transfusion.

*SD*, standard deviation.

### Subgroup analyses

**Figure 3:**
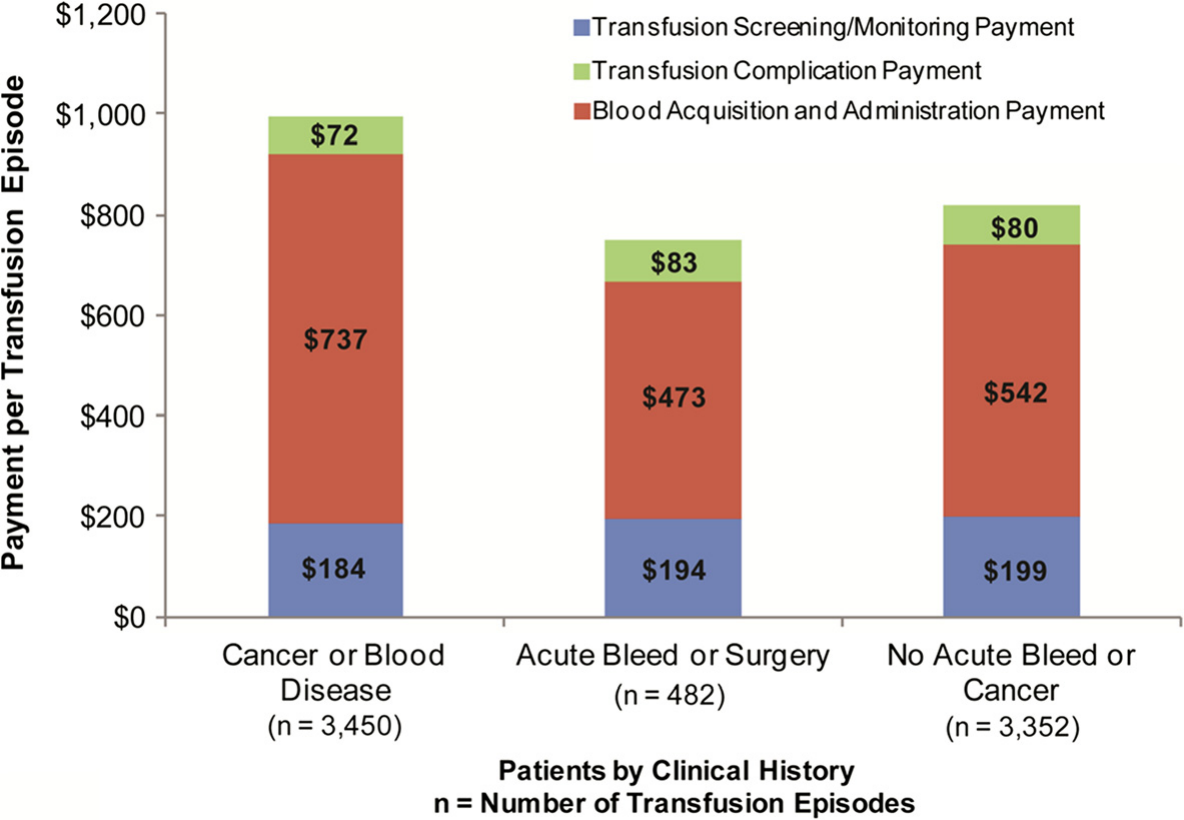
Red blood cell transfusion episode payments for patients with history of acute bleed/surgery or cancer/blood diseases.

We estimated per RBC transfusion episode payments for 3 subgroups (Figure 3). Screening and monitoring costs varied minimally between patients with cancer or blood disease; patients with an acute bleed or surgery; and patients who did not have cancer, blood disease, acute bleed, or surgery. However, there was significant variation in blood acquisition and administration payments. Patients with cancer or blood disease had the highest mean payment (mean, $737; SD, $1,502), followed by patients with neither acute bleed nor cancer (mean, $542; SD, $1,044). Mean total payment per RBC transfusion episode was higher than base-case estimates (Table 2) for patients with cancer or blood disease (mean, $969; SD, $1,948) and lower than base-case estimates for patients with an acute bleed or surgery (mean, $733; SD, $1,195).

**Figure 4:**
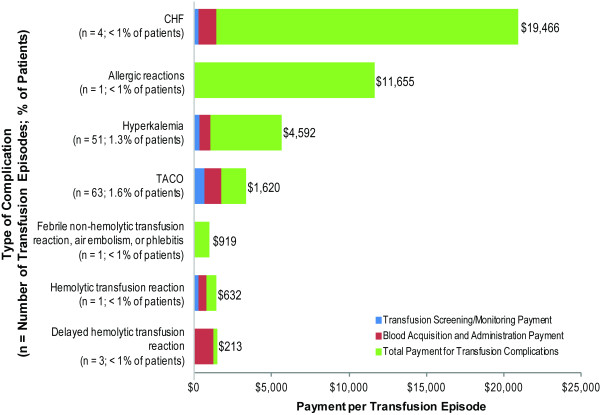
**Mean red blood cell transfusion payments for patients with complications, by type of complication.** Abbreviations: CHF, congestive heart failure; TACO, transfusion-associated circulatory overload.

Figure 4 summarizes payments made for various types of transfusion-related complications. Payments for CHF (mean, $19,466) and allergic reactions (mean, $11,655) were the most expensive complication payments. TACO (63 episodes) and hyperkalemia (51 episodes), were the most commonly observed types of complications. Among the RBC transfusion episodes associated with hyperkalemia complications, 69.1% of the hyperkalemia events occurred on the same date as the RBC transfusion start date.

### Sensitivity analyses

We performed sensitivity analyses by excluding cost outliers, varying time frames (narrow- and broad-window time frames) and reestimating the component and total payments per RBC transfusion episode. As shown in Table 3, relative to base-case estimates, screening and monitoring component payments increased slightly when outliers were excluded (mean, $214; SD, $451) and when we used the broad-window time frame (mean, $225; SD, $712) but decreased slightly when we used the narrow-window time frame (mean, $172; SD, $598). Blood acquisition and administration payments were higher when outliers were excluded (mean, $696; SD, $701) but similar to the base case estimates when narrowing and broadening the time frame. When using the broad-window time frame, complication payments greatly increased from the base case’s mean (SD) of $75 ($1,317) to a mean of $120 ($1,520). Total payment per RBC transfusion episode was highest when cost outliers were excluded (mean, $971; SD, $1,982) and when we used the broad-window time frame (mean, $931; SD, $2,239) compared to the base-case estimates.

**Table 3:** Sensitivity analysis of RBC transfusion episode payments by time frame

**Mean payments (SD)**	**Base case**	**Drop top 1% and bottom $0 patients**	**Narrow window**	**Broad window**
	**(N = 7,049)**	**(N = 4,844)**	**(N = 7,049)**	**(N = 7,049)**
Screening/monitoring	$193 (615)	$214 (451)	$172 (598)	$225 (712)
Acquisition/administration	$615 (1,237)	$696 (701)	$615 (1,237)	$615 (1,237)
Complication	$75 (1,317)	$86 (1,541)	$56 (1,149)	$120 (1,521)
**Total payment per RBC transfusion**	**$854 (2,060)**	**$971 (1,982)**	**$814 (1,940)**	**$931 (2,239)**

*RBC*, red blood cell; *SD*, standard deviation.

Finally, we estimated component payments per blood acquisition and administration unit. Per-unit data were available for 340 outpatient RBC transfusion episodes. When these episodes were analyzed, the transfusion screening/monitoring mean (SD) payment was $245 ($425), the blood acquisition and administration mean payment was $433 ($495), and the mean payment for transfusion complications was $153 ($1,915). Per-unit total transfusion episode mean payment was $827 ($2,127).

## Discussion

We evaluated 3,283 chronic dialysis patients with at least 1 outpatient setting RBC transfusion episode. Most of the patients had diabetes measured during the pre-index period. In the base case, 72.1% of the total payments were due to blood acquisition and administration, with the remainder of payments attributable to screening and monitoring and, to a lesser extent, transfusion-related complications. Total RBC transfusion payments were higher for patients with cancer or blood disease than those with an acute bleed or in our base-case estimates. Sensitivity analyses suggest that the base-case results were robust. Total payment estimates increased when both the top 1% most expensive episodes and $0 payment episodes were excluded. Payments became slightly lower when a narrow-window time frame was used and increased slightly with a broad-window time frame. The variations in total payments among all sensitivity analyses were less than 15% different from the base-case total payment estimates. Across all sensitivity analyses, the blood acquisition and administration payments consistently were the most expensive component payment.

Under the newly implemented Medicare PPS for ESRD patients, reimbursement is capitated to include dialysis and previously separately billable medications and services. Payments for blood and blood products are not, however, included in the new PPS bundle. As patients are treated to a lower hemoglobin level, the resulting lower hemoglobin levels could also create a medical necessity for RBC transfusions. The use of transfusions to supplement ESA therapy in ESRD patients on dialysis may increase because of economic incentives and clinical necessity. It is, therefore, important to comprehensively examine the transfusion-associated payments made within the chronic dialysis patient population in order to understand the economic consequences of the recent changes in reimbursement.

Our results differ somewhat from a recent cost analysis that used an activity-based costing model of RBC transfusions in a surgical population (based on observation of real-life activities in four hospitals in the United States and Europe) to identify the costs for each transfusion-related task and resource [21]. Overall, total inpatient RBC transfusion costs were $522 to $1183 (mean, $761) per unit across the four hospitals (a considerable portion of the costs was related to pre-surgical testing for blood type/screening in patients who never received a transfusion) [21]. These results were similar but slightly lower than our mean per-unit payment estimate of $827 (SD, $2,127). Blood acquisition costs in the other study were only 21% to 32% ($154 to $248 in 2008 dollars) of the total RBC transfusion-related costs. We found blood acquisition and administration payments accounted for 50% to70% of total payments, but could not differentiate acquisition and administration payments with certainty in the claims data. Patient testing and administration and monitoring of RBC transfusions and pretransfusion processes were 24% to 36% of total costs. Managing acute transfusion reactions and hemovigilance contributed to 0% to 2% of costs [21]. The type and level of detail available in the data as well as place of service (inpatient vs outpatient setting) may explain some of the differences in our estimates from those of Shandler et al. Moreover, costs associated with blood acquisition and administration can vary according to the amount (units) and type (eg, leukoreduced or irradiated) of blood.

Our study had several limitations. We followed patients for mean 494.76 days, which was not long enough to detect payments for iron overload. The analysis took a conservative approach and excludes a number of potential resources that may increase the potential economic burden. For example, a number of potential long-term complications, including infectious diseases and iron overload, were excluded and may result in an underestimation of the overall economic burden of RBC transfusions. In addition, long-term management of acute complications such as medication costs and additional outpatient management were not included in the analysis, all of which may result in underestimation of the overall economic burden of RBC transfusions. Lastly, the economic burden may be underestimated because only hospitalizations related to specific complications listed in Figure 2 were included. The analysis may be potentially underestimating the economic burden by not including hospitalization that may occur the day of or day after a RBC transfusion because this may be a result of the transfusion exacerbating a existing condition or producing a new condition. Another limitation is the potential to include acute renal failure patients in the analysis. Utilizing a large number of dialysis claims over a long period may result in a much healthier population as a result of inclusion criteria. To minimize the potential for including acute renal failure or only including a healthier population of ESRD patients on dialysis, the analysis presented here utilizes specific codes that are only utilized by ESRD patients on dialysis. Another includes hospitalizations related to the specific acute complication events included in the micro-costing design. We did not have information on patient race/ethnicity. We were missing type of dialysis in about one third of cases. We did not evaluate inpatient costs because the inpatient claims data were based on diagnosis-related group (DRG) codes and could not be separated out into payment components. The focus of this analysis was on the payer burden of RBC transfusions and as a result of payments for inpatient hospital admissions being capitated into DRG payments, there is no ability to estimate the payer burden of inpatient-administered RBC transfusions. The majority (about 85%) of transfusions for dialysis patients occur in the inpatient setting [22], and thus the economic burden of transfusions is likely greatest in the inpatient setting, but of concern to the inpatient hospital rather than the third party payer, which is beyond the scope of this analysis. The economic burden of inpatient transfusions is likely to be similar to those for outpatient transfusions and the costs associated with complications arising from outpatient transfusions are also likely to be similar, if not greater than outpatient transfusions as a result of patient severity (as demonstrated by the patient being in the inpatient setting). Future analyses should focus on the provider cost burden of both outpatient and inpatient administered RBC transfusions. Finally, patients in our sample had either commercial insurance or Medicare plus Medicare supplemental insurance as their primary coverage, and therefore, the results may not be generalizable to patients who are uninsured, are covered only by Medicare, or have other types of insurance coverage.

## Conclusion

This is the first study to examine payments for outpatient RBC transfusions in a population of patients undergoing chronic dialysis. Our study shows that payments for outpatient RBC transfusion episodes are primarily driven by blood acquisition and administration payments. Additionally, there are travel and other costs to dialysis patients for RBC transfusion episodes and increased risk for allosensitization; these could not be estimated here, but are important costs associated with RBC transfusions. While infrequent, transfusion complications increase payments substantially when they occur. Better understanding of RBC transfusion episodes’ payments and costs to patient may help inform policy makers when determining the appropriate reimbursement policy for chronic dialysis patients.

## Abbreviations

CHF: Congestive heart failure; CPT: Current Procedural Terminology; DOPPS: Dialysis Outcomes and Practice Patterns Study; ESA: Erythropoiesis-stimulating agent; ESRD: End-stage renal disease; HCPCS: Healthcare Common Procedure Coding System; PPS: Prospective Payment System; RBC: Red blood cell; SD: Standard deviation; TACO: Transfusion-associated circulatory overload; TRALI: Transfusion-related acute lung injury; USRDS: United States Renal Data System.

## Competing interests

Dr. Spiegel has been a consultant, received research funding, and served on the speakers bureau for Amgen, and has served on scientific advisory boards for Amgen and Genzyme. Dr. Carson financial research support from Amgen. Dr. Custer received consulting fees from Amgen for this project. Dr. Song, Dr. Cao, Dr. Cappell, and Ms. Varker are employees of Truven Health, Cambridge, MA, which provides consulting services to clients, including the pharmaceutical industry. Dr. Gitlin, Dr. Lee, Dr. Wan, and Dr. Ashfaq are employees and stockholders of Amgen Inc.

## Authors' contributions

MG contributed to the conception of the study, study design, analysis and interpretation of the data; contributed to the writing and revision of the manuscript; and approved the final draft for submission. JAL contributed to the conception of the study, study design, analysis and interpretation of the data; contributed to the writing and revision of the manuscript; and approved the final draft for submission. DMS contributed to the conception of the study, study design, analysis and interpretation of the data; contributed to the writing and revision of the manuscript; and approved the final draft for submission. JLC contributed to the conception of the study, study design, analysis and interpretation of the data; contributed to the writing and revision of the manuscript; and approved the final draft for submission. BSC contributed to the conception of the study, study design, analysis and interpretation of the data; contributed to the writing and revision of the manuscript; and approved the final draft for submission. SW contributed to the conception of the study, study design, analysis and interpretation of the data; contributed to the writing and revision of the manuscript; and approved the final draft for submission. AA contributed to the conception of the study, study design, analysis and interpretation of the data; contributed to the writing and revision of the manuscript; and approved the final draft for submission. XS contributed to acquisition of data and analysis and interpretation of the data, contributed to the writing and revision of the manuscript, and approved the final draft for submission. ZC contributed to acquisition of data and analysis and interpretation of the data, contributed to the writing and revision of the manuscript, and approved the final draft for submission. KAC contributed to acquisition of data and analysis and interpretation of the data, contributed to the writing and revision of the manuscript, and approved the final draft for submission. HVV contributed to the acquisition of the data, contributed to the writing and revision of the manuscript, and approved the final draft for submission.

## Pre-publication history

The pre-publication history for this paper can be accessed here:

http://www.biomedcentral.com/1471-2369/13/145/prepub

## Supplementary Material

Additional file 1 Supplementary Material: Inpatient and Outpatient Transfusion Billing Codes. Click here for file

## References

[B1] Sargent JA Acchiardo SR Iron requirements in hemodialysis Blood Purif 2004 22 1 112 123 10.1159/000074931 14732819

[B2] Churchill DN Taylor DW Cook RJ LaPlante P Barre P Cartier P Fay WP Goldstein MB Jindal K Mandin H Canadian hemodialysis morbidity study Am J Kidney Dis 1992 19 3 214 234 1553966 10.1016/s0272-6386(13)80002-9

[B3] Eschbach JW Adamson JW Anemia of end-stage renal disease (ESRD) Kidney Int 1985 28 1 1 5 10.1038/ki.1985.109 3900528

[B4] Beauregard P Blajchman MA Hemolytic and pseudo-hemolytic transfusion reactions: an overview of the hemolytic transfusion reactions and the clinical conditions that mimic them Transfus Med Rev 1994 8 3 184 199 10.1016/S0887-7963(94)70110-3 8081080

[B5] Despotis GJ Zhang L Lublin DM Transfusion risks and transfusion-related pro-inflammatory responses Hematol Oncol Clin North Am 2007 21 1 147 161 10.1016/j.hoc.2006.11.002 17258124 PMC7135740

[B6] Dodd RY Notari EP Stramer SL Current prevalence and incidence of infectious disease markers and estimated window-period risk in the American Red Cross blood donor population Transfusion (Paris) 2002 42 8 975 979 10.1046/j.1537-2995.2002.00174.x 12385406

[B7] Eder AF Chambers LA Noninfectious complications of blood transfusion Arch Pathol Lab Med 2007 131 5 708 718 17488156 10.5858/2007-131-708-NCOBT

[B8] Gilliss BM Looney MR Gropper MA Reducing noninfectious risks of blood transfusion Anesthesiology 2011 115 3 635 649 10.1097/ALN.0b013e31822a22d9 21792054 PMC3162102

[B9] Simon GE Bove JR The potassium load from blood transfusion Postgrad Med 1971 49 6 61 64 10.1080/00325481.1971.11696651 5162365

[B10] Vella JP O’Neill D Atkins N Donohoe JF Walshe JJ Sensitization to human leukocyte antigen before and after the introduction of erythropoietin Nephrol Dial Transplant 1998 13 8 2027 2032 10.1093/ndt/13.8.2027 9719159

[B11] The 2009 National Blood Collection and Utilization Survey Report 2011 Washington, DC: US Department of Health and Human Services, Office of the Assistant Secretary for Health

[B12] Allain JP Stramer SL Carneiro-Proietti AB Martins ML da Silva SN L Ribeiro M Proietti FA Reesink HW Transfusion-transmitted infectious diseases Biologicals 2009 37 2 71 77 10.1016/j.biologicals.2009.01.002 19231236

[B13] Healthcare Cost and Utilization Project (HCUP) HCUP Nationwide Inpatient Sample (NIS) 2007–2009 Rockville, MD: Agency for Healthcare Research and Quality 21413206

[B14] Kumar A Perioperative management of anemia: limits of blood transfusion and alternatives to it Cleve Clin J Med 2009 76 Suppl 4 S112 S118 19880828 10.3949/ccjm.76.s4.18

[B15] Goodnough LT Strasburg D Riddell J Verbrugge D Wish J Has recombinant human erythropoietin therapy minimized red-cell transfusions in hemodialysis patients? Clin Nephrol 1994 41 5 303 307 8050211

[B16] Ibrahim HN Ishani A Foley RN Guo H Liu J Collins AJ Temporal trends in red blood transfusion among US dialysis patients, 1992–2005 Am J Kidney Dis 2008 52 6 1115 1121 10.1053/j.ajkd.2008.07.022 18823686

[B17] US Renal Data System USRDS 1995 Annual Data Report 1995 Bethesda, MD: The National Institutes of Health, National Institute of Diabetes and Digestive and Kidney Diseases

[B18] Pisoni RL Bragg-Gresham JL Young EW Akizawa T Asano Y Locatelli F Bommer J Cruz JM Kerr PG Mendelssohn DC Anemia management and outcomes from 12 countries in the Dialysis Outcomes and Practice Patterns Study (DOPPS) Am J Kidney Dis 2004 44 1 94 111 10.1053/j.ajkd.2004.03.023 15211443

[B19] Drummond MF O’Brien B Stoddart GL Torrance GW Methods for the economic-evaluation of health care programmes 1997 New York, NY: Oxford University Press

[B20] Gold MR Siegel JE Russell LB Weinstein MC Cost-Effectiveness in Health and Medicine 1996 New York, NY: Oxford University Press

[B21] Shander A Hofmann A Ozawa S Theusinger OM Gombotz H Spahn DR Activity-based costs of blood transfusions in surgical patients at four hospitals Transfusion (Paris) 2010 50 4 753 765 10.1111/j.1537-2995.2009.02518.x 20003061

[B22] Lawler EV Bradbury BD Fonda JR Gaziano JM Gagnon DR Transfusion burden among patients with chronic kidney disease and anemia Clin J Am Soc Nephrol 2010 5 4 667 672 10.2215/CJN.06020809 20299366 PMC2849699

