# Supplementary material for: Outpatient red blood cell transfusion payments among patients on chronic dialysis
Source: BMC Nephrol. 2012 Nov 2;13:145. doi: 10.1186/1471-2369-13-145 (PMC3532082; doi:10.1186/1471-2369-13-145)
Supplement: Additional file 1 — Supplementary Material: Inpatient and Outpatient Transfusion Billing Codes. [file 1471-2369-13-145-S1.docx]

**Supplementary Material: Inpatient and Outpatient Transfusion Billing Codes**

**Table S1.** Codes for Chronic Dialysis Claims

| **Code Type** | **Code** | **Description** |
| --- | --- | --- |
| UB-02 (Rev Code) | 821 | Hemodialysis/composite or other rate |
| UB-02 (Rev Code) | 831 | Peritoneal/composite or other rate |
| UB-02 (Rev Code) | 841 | CAPD/composite or other rate |
| UB-02 (Rev Code) | 851 | CCPD/composite or other rate |
| CPT | 90918 | corresponds to HCPCS G0308-G0310, G0320 |
| CPT | 90919 | corresponds to HCPCS G0311-G0313, G0321 |
| CPT | 90920 | corresponds to HCPCS G0314-G0316, G0322 |
| CPT | 90921 | corresponds to HCPCS G0317-G0319, G0323 |
| CPT | 90922 | corresponds to HCPCS G0324 |
| CPT | 90923 | corresponds to HCPCS G0325 |
| CPT | 90924 | corresponds to HCPCS G0326 |
| CPT | 90925 | corresponds to HCPCS G0327 |
| HCPCS | G0308 | End stage renal disease (ESRD) related services during the course of treatment, for patients under 2 years of age to include monitoring for the adequacy of nutrition, assessment of growth and development, and counseling of parents; with 4 or more face-to-face physician visits per month |
| HCPCS | G0309 | End stage renal disease (ESRD) related services during the course of treatment for patients under 2 years of age to include monitoring for the adequacy of nutrition, assessment of growth and development, and counseling of parents; with 2 or 3 face-to-face physician visits per month |
| HCPCS | G0310 | End stage renal disease (ESRD) related services during the course of treatment, for patients under 2 years of age to include monitoring for the adequacy of nutrition, assessment of growth and development, and counseling of parents; with 1 face-to-face physician visit per month |
| HCPCS | G0311 | End stage renal disease (ESRD) related services during the course of treatment, for patients between 2 and 11 years of age to include monitoring for the adequacy of nutrition, assessment of growth and development, and counseling of parents; with 4 or more face-to-face physician visits per month |
| HCPCS | G0312 | End stage renal disease (ESRD) related services during the course of treatment, for patients between 2 and 11 years of age to include monitoring for the adequacy of nutrition, assessment of growth and development, and counseling of parents; with 2 or 3 face-to-face physician visits per month |
| HCPCS | G0313 | End stage renal disease (ESRD) related services during the course of treatment, for patients between 2 and 11 years of age to include monitoring for the adequacy of nutrition, assessment of growth and development, and counseling of parents; with 1 face-to-face physician visit per month |
| HCPCS | G0314 | End stage renal disease (ESRD) related services, during the course of treatment, for patients between 12 and 19 years of age to include monitoring for the adequacy of nutrition, assessment of growth and development, and counseling of parents; with 4 or more face-to-face physician visits per month |
| HCPCS | G0315 | End stage renal disease (ESRD) related services during the course of treatment, for patients between 12 and 19 years of age to include monitoring for the adequacy of nutrition, assessment of growth and development, and counseling of parents; with 2 or 3 face-to-face physician visits per month |
| HCPCS | G0316 | End stage renal disease (ESRD) related services during the course of treatment, for patients between 12 and 19 years of age to include monitoring for the adequacy of nutrition, assessment of growth and development, and counseling of parents; with 1 face-to-face physician visit per month |
| HCPCS | G0317 | End stage renal disease (ESRD) related services during the course of treatment, for patients **20 years** **of age and over**; with 4 or more face-to-face physician visits per month |
| HCPCS | G0318 | End stage renal disease (ESRD) related services during the course of treatment, for patients **20 years of age and over**; with 2 or 3 face-to-face physician visits per month |
| HCPCS | G0319 | End stage renal disease (ESRD) related services during the course of treatment, for patients **20 years of age and over**; with 1 face-to-face physician visit per month |
| HCPCS | G0320 | End stage renal disease (ESRD) related services for home dialysis patients per full month; for patients **under two years** of age to include monitoring for adequacy of nutrition, assessment of growth and development, and counseling of parents |
| HCPCS | G0321 | End stage renal disease (ESRD) related services for home dialysis patients per full month; for patients **two to eleven** years of age to include monitoring for adequacy of nutrition, assessment of growth and development, and counseling of parents |
| HCPCS | G0322 | End stage renal disease (ESRD) related services for home dialysis patients per full month; for patients **twelve to nineteen** years of age to include monitoring for adequacy of nutrition, assessment of growth and development, and counseling of parents |
| HCPCS | G0323 | End stage renal disease (ESRD) related services for home dialysis patients per full month; for patients **twenty years of age and older** |
| HCPCS | G0324 | End stage renal disease (ESRD) related services less than full month, per day; for patients **under two** years of age |
| HCPCS | G0325 | End stage renal disease (ESRD) related services less than full month, per day; for patients **between two and eleven** years of age |
| HCPCS | G0326 | End stage renal disease (ESRD) related services less than full month, per day; for patients **between twelve and nineteen** years of age |
| HCPCS | G0327 | End stage renal disease (ESRD) related services less than full month, per day; for patients **twenty years of age and over** |
| CPT | 90951 | End-stage renal disease (ESRD) related services monthly, for patients younger than 2 years of age to include monitoring for the adequacy of nutrition, assessment of growth and development, and counseling of parents; with 4 or more face-to-face physician visits per month |
| CPT | 90952 | End-stage renal disease (ESRD) related services monthly, for patients younger than 2 years of age to include monitoring for the adequacy of nutrition, assessment of growth and development, and counseling of parents; with 2-3 face-to-face physician visits per month |
| CPT | 90953 | End-stage renal disease (ESRD) related services monthly, for patients younger than 2 years of age to include monitoring for the adequacy of nutrition, assessment of growth and development, and counseling of parents; with 1 face-to-face physician visit per month |
| CPT | 90954 | End-stage renal disease (ESRD) related services monthly, for patients 2-11 years of age to include monitoring for the adequacy of nutrition, assessment of growth and development, and counseling of parents; with 4 or more face-to-face physician visits per month |
| CPT | 90955 | End-stage renal disease (ESRD) related services monthly, for patients 2-11 years of age to include monitoring for the adequacy of nutrition, assessment of growth and development, and counseling of parents; with 2-3 face-to-face physician visits per month |
| CPT | 90956 | End-stage renal disease (ESRD) related services monthly, for patients 2-11 years of age to include monitoring for the adequacy of nutrition, assessment of growth and development, and counseling of parents; with 1 face-to-face physician visit per month |
| CPT | 90957 | End-stage renal disease (ESRD) related services monthly, for patients 12-19 years of age to include monitoring for the adequacy of nutrition, assessment of growth and development, and counseling of parents; with 4 or more face-to-face physician visits per month |
| CPT | 90958 | End-stage renal disease (ESRD) related services monthly, for patients 12-19 years of age to include monitoring for the adequacy of nutrition, assessment of growth and development, and counseling of parents; with 2-3 face-to-face physician visits per month |
| CPT | 90959 | End-stage renal disease (ESRD) related services monthly, for patients 12-19 years of age to include monitoring for the adequacy of nutrition, assessment of growth and development, and counseling of parents; with 1 face-to-face physician visit per month |
| CPT | 90960 | End-stage renal disease (ESRD) related services monthly, for patients 20 years of age and older; with 4 or more face-to-face physician visits per month |
| CPT | 90961 | End-stage renal disease (ESRD) related services monthly, for patients 20 years of age and older; with 2-3 face-to-face physician visits per month |
| CPT | 90962 | End-stage renal disease (ESRD) related services monthly, for patients 20 years of age and older; with 1 face-to-face physician visit per month |
| CPT | 90963 | End-stage renal disease (ESRD) related services for home dialysis per full month, for patients younger than 2 years of age to include monitoring for the adequacy of nutrition, assessment of growth and development, and counseling of parents |
| CPT | 90964 | End-stage renal disease (ESRD) related services for home dialysis per full month, for patients 2-11 years of age to include monitoring for the adequacy of nutrition, assessment of growth and development, and counseling of parents |
| CPT | 90965 | End-stage renal disease (ESRD) related services for home dialysis per full month, for patients 12-19 years of age to include monitoring for the adequacy of nutrition, assessment of growth and development, and counseling of parents |
| CPT | 90966 | End-stage renal disease (ESRD) related services for home dialysis per full month, for patients 20 years of age and older |
| CPT | 90967 | End-stage renal disease (ESRD) related services for dialysis less than a full month of service, per day; for patients younger than 2 years of age |
| CPT | 90968 | End-stage renal disease (ESRD) related services for dialysis less than a full month of service, per day; for patients 2-11 years of age |
| CPT | 90969 | End-stage renal disease (ESRD) related services for dialysis less than a full month of service, per day; for patients 12-19 years of age |
| CPT | 90970 | End-stage renal disease (ESRD) related services for dialysis less than a full month of service, per day; for patients 20 years of age and older |

**Table S2.** Billing Codes for Transfusions Administered in the Outpatient Setting

| **Code Type** | **Code** | **Description** |
| --- | --- | --- |
| UB-04 | 0390 | General Classification: Blood Processing and Services |
| UB-04 | 0391 | Blood Administration |
| UB-04 | 0392 | Blood Processing and Storage |
| UB-04 | 0399 | Other Blood Handling |
| UB-04 | 0380 | General Classification: Blood Products |
| UB-04 | 0381 | Packed Red Cells |
| CPT | 36430 | Transfusion, blood or blood components |
| CPT | P9011 | Blood (split unit), specify amount |
| CPT | P9016 | Red blood cells, leukocytes reduced, each unit |
| CPT | P9021 | Red blood cells, each unit |
| CPT | P9022 | Red blood cells, washed, each unit |
| CPT | P9038 | Red blood cells , irradiated, each unit |
| CPT | P9039 | Red blood cells, deglycerolized, each unit |
| CPT | P9040 | Red blood cells, leukocytes reduced, irradiated, each unit |
| CPT | P9051 | Whole blood or red blood cells, leukocytes reduced |
| CPT | P9054 | Whole blood or red blood cells, leukocytes reduced, frozen, deglycerol, washed, each unit |
| CPT | P9057 | Red blood cells, frozen/deglycerolized/washed, leukocytes reduced, irradiated, each unit |
| CPT | P9058 | Red blood cells, leukocytes reduced, CMV-negative, irradiated, each unit |

Abbreviations: CMV, cytomegalovirus; CPT, current procedural terminology.

**Table S3:** Diagnosis Codes for Transfusion-Related Complications in the Outpatient Setting

| **Code Type** | **Code** | **Category** | **Description** |
| --- | --- | --- | --- |
| ICD-9-CM Dx | 398.91 | CHF | Rheumatic (congestive) heart failure |
| ICD-9-CM Dx | 402.x1 | CHF | Hypertensive heart disease with heart failure |
| ICD-9-CM Dx | 404.x1 | CHF | Hypertensive heart and chronic kidney disease with heart failure and with chronic kidney disease stage I through stage IV, or unspecified |
| ICD-9-CM Dx | 404.x3 | CHF | Hypertensive heart and chronic kidney disease with heart failure and chronic kidney disease stage V or end stage renal disease |
| ICD-9-CM Dx | 428.xx | CHF | Heart failure |
| ICD-9-CM Dx | 708.0 | Allergic reaction | Allergic urticaria |
| ICD-9-CM Dx | 995.0 | Allergic reaction | Other anaphylactic shock |
| ICD-9-CM Dx | 999.4 | Allergic reaction | Anaphylactic shock due to serum |
| ICD-9-CM Dx | 999.83 | Hemolytic transfusion reaction | Hemolytic transfusion reaction, incompatibility unspecified |
| ICD-9-CM Dx | 999.84 | Hemolytic transfusion reaction | Acute hemolytic transfusion reaction, incompatibility unspecified |
| ICD-9-CM Dx | 999.85 | Hemolytic transfusion reaction | Delayed hemolytic transfusion reaction, incompatibility unspecified |
| ICD-9-CM Dx | 999.6x | Hemolytic transfusion reaction | Hemolytic transfusion reactions |
| ICD-9-CM Dx | 999.7x | Hemolytic transfusion reaction | Hemolytic transfusion reactions |
| ICD-9-CM Dx | 276.7 | Hyperkalemia | Hyperkalemia |
| ICD-9-CM Dx | 999.1 | Febrile nonhemolytic transfusion reaction, air embolism or phlebitis | Other, including febrile nonhemolytic transfusion reaction, air embolism or phlebitis following transfusion |
| ICD-9-CM Dx | 999.2 | Febrile nonhemolytic transfusion reaction, air embolism or phlebitis | Other, including febrile nonhemolytic transfusion reaction, air embolism or phlebitis following transfusion |
| ICD-9-CM Dx | 999.80 | Febrile nonhemolytic transfusion reaction, air embolism or phlebitis | Transfusion reaction, unspecified |
| ICD-9-CM Dx | 999.89 | Febrile nonhemolytic transfusion reaction, air embolism or phlebitis | Other, including febrile nonhemolytic transfusion reaction, air embolism or phlebitis following transfusion |
| ICD-9-CM Dx | E934.7 | Febrile nonhemolytic transfusion reaction, air embolism or phlebitis | Other, including febrile nonhemolytic transfusion reaction, air embolism or phlebitis following transfusion |
| ICD-9-CM Dx | 276.6x | TACO | Fluid overload disorder |
| ICD-9-CM Dx | 518.7 | TRALI | Transfusion-associated acute lung injury (TRALI) |

Abbreviations: CHF, congestive heart failure; TACO, transfusion-associated circulatory overload; TRALI, transfusion-related acute lung injury.
